# Bis[2-(2,4-dinitro­benz­yl)pyridinium] biphenyl-4,4′-disulfonate trihydrate

**DOI:** 10.1107/S1600536810014819

**Published:** 2010-04-28

**Authors:** Graham Smith, Urs D. Wermuth, David J. Young

**Affiliations:** a Faculty of Science and Technology, Queensland University of Technology, GPO Box 2434, Brisbane, Queensland 4001, Australia; b School of Biomolecular and Physical Sciences, Griffith University, Nathan, Queensland 4111, Australia

## Abstract

In the structure of the title salt, 2C_12_H_10_N_3_O_4_^+^·C_12_H_8_O_6_S_2_^2−^·3H_2_O, determined at 173 K, the biphenyl-4,4′-disulfonate dianions lie across crystallographic inversion centres with the sulfonate groups inter­acting head-to-head through centrosymmetric cyclic bis­(water)-bridged hydrogen-bonding associations [graph set *R*_4_^4^(11)], forming chains. The 2-(2,4-dinitro­benz­yl)pyridinium cations are linked to these chains through pyridinium–water N—H⋯O hydrogen bonds and a two-dimensional network is formed through water bridges between sulfonate and 2-nitro O atoms, while the structure also has weak cation–anion π–π aromatic ring inter­actions [minimum ring centroid separation = 3.8441 (13) Å].

## Related literature

For structural data on 2-(2,4-dinitro­benz­yl)pyridine and related compounds, see Seff & Trueblood (1968 ▶); Scherl *et al.* (1996 ▶); Naumov *et al.* (2002 ▶, 2005 ▶). For bipyridine-4,4′-disulfonate compounds, see: Swift *et al.* (1998 ▶); Swift & Ward (1998 ▶); Holman & Ward (2000 ▶); Liao *et al.* (2001 ▶). For graph-set notation, see: Etter *et al.* (1990 ▶).
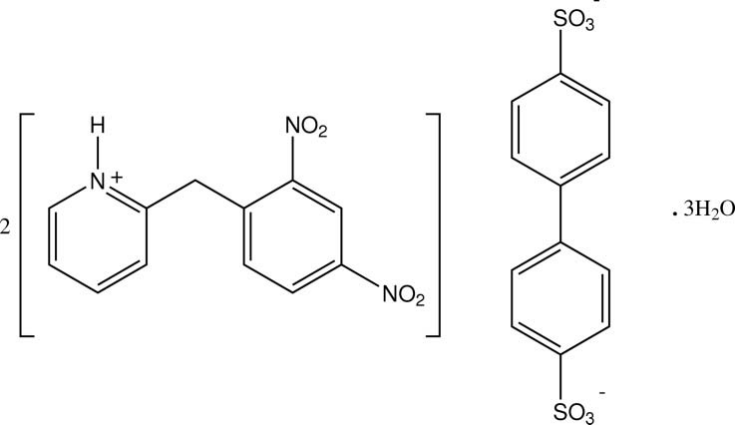


## Experimental

### 

#### Crystal data

2C_12_H_10_N_3_O_4_^+^·C_12_H_8_O_6_S_2_^2−^·3H_2_O
*M* *_r_* = 886.83
Triclinic, 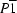

*a* = 8.3897 (3) Å
*b* = 10.6455 (4) Å
*c* = 11.7405 (5) Å
α = 97.879 (3)°
β = 96.926 (3)°
γ = 112.066 (4)°
*V* = 945.53 (7) Å^3^
*Z* = 1
Mo *K*α radiation
μ = 0.23 mm^−1^
*T* = 173 K
0.30 × 0.25 × 0.15 mm

#### Data collection

Oxford Diffraction Gemini-S CCD-detector diffractometer
Absorption correction: multi-scan (*SADABS*; Sheldrick, 1996 ▶) *T*_min_ = 0.98, *T*_max_ = 0.99
8964 measured reflections
3844 independent reflections
3441 reflections with *I* > 2σ(*I*)
*R*_int_ = 0.020

#### Refinement

*R*[*F* ^2^ > 2σ(*F* ^2^)] = 0.042
*wR*(*F* ^2^) = 0.104
*S* = 1.03
3844 reflections
296 parameters
H atoms treated by a mixture of independent and constrained refinement
Δρ_max_ = 0.35 e Å^−3^
Δρ_min_ = −0.30 e Å^−3^

### 

Data collection: *CrysAlis CCD* (Oxford Diffraction, 2008 ▶); cell refinement: *CrysAlis RED* (Oxford Diffraction, 2008 ▶); data reduction: *CrysAlis RED*; program(s) used to solve structure: *SIR92* (Altomare *et al.*, 1994 ▶); program(s) used to refine structure: *SHELXL97* (Sheldrick, 2008 ▶) within *WinGX* (Farrugia, 1999 ▶); molecular graphics: *PLATON* (Spek, 2009 ▶); software used to prepare material for publication: *PLATON*.

## Supplementary Material

Crystal structure: contains datablocks global, I. DOI: 10.1107/S1600536810014819/ng2763sup1.cif

Structure factors: contains datablocks I. DOI: 10.1107/S1600536810014819/ng2763Isup2.hkl

Additional supplementary materials:  crystallographic information; 3D view; checkCIF report

## Figures and Tables

**Table 1:** Hydrogen-bond geometry (Å, °)

*D*—H⋯*A*	*D*—H	H⋯*A*	*D*⋯*A*	*D*—H⋯*A*
N1—H1⋯O1*W*	0.95 (3)	1.71 (3)	2.655 (3)	175 (3)
O1*W*—H11*W*⋯O43*A*^i^	0.88 (4)	1.84 (4)	2.716 (2)	175 (3)
O1*W*—H12*W*⋯O41*A*	0.80 (3)	2.01 (3)	2.806 (2)	172 (3)
O2*W*—H21*W*⋯O43*A*	0.82 (4)	1.99 (4)	2.761 (4)	155 (4)
O2*W*—H22*W*⋯O21^ii^	0.87 (3)	2.32 (3)	2.867 (2)	124 (3)

Symmetry codes: (i) 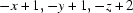; (ii) 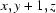.

**Fig. 1.**
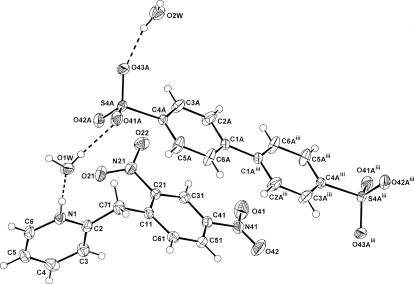
Molecular configuration and atom naming scheme for the DNBP cation, the BPDS dianion and the two water molecules of solvation [O1W , O2W, with the latter having SOF = 0.5 (1)], in the asymmetric unit of (I). The dianion lies across an inversion centre [symmetry code (iii) -x + 2, -y + 1, -z +1] and displacement ellipsoids are drawn at the 50% probability level.

**Fig. 2.**
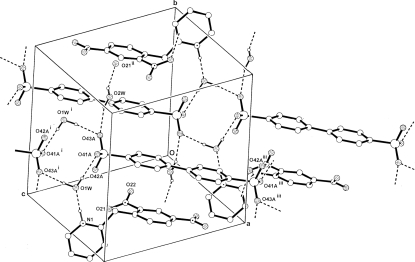
The two-dimensional hydrogen-bonded network structure of (I) extending viewed down the approximate a cell direction showing the water-linked BPDS chains and water-bridged extensions down b. Hydrogen bonds are shown as dashed lines and non-interactive H atoms are omitted. For symmetry codes, see Table 1.

## supplementary crystallographic information

### Comment

The Lewis base 2-(2,4-dinitrobenzyl)pyridine (DNBP) has been a compound of considerable interest for more than 40 years because of its unusual photochromic characteristics. Irradiation of the colourless crystals with light of wavelength 400nm or less results in the formation of a deep blue coloration in a reversible tautomeric reaction. The structure of the colourless form has been determined (Seff & Trueblood, 1968; Scherl et al., 1996), while in another determination (Naumov et al., 2002), the structures of both forms were determined, confirming the presence of two-photon excitation giving nitro-assisted proton transfer (NAPT) involving an oxygen of the o-nitro substituent group. The effect is not present in the p-nitro-substituted isomer. Although the structure of the chloride salt of DNBP is known (Naumov et al., 2005), no other examples of analogous compounds are present in the CSD.

Of a number of reactions of DNBP with aromatic carboxylic and sulfonic acids in 50% ethanol–water, we found that only one, biphenyl-4,4'-disulfonic acid (BPDS) gave crystals of suitable quality for X-ray analysis, the title compound 2(C_12_H_10_N_3_O_4_^+^)  C_12_H_8_O_6_S_2_^2-^ . 3H_2_O (I), the structure of which is reported here. The structures of 1:2 proton-transfer compounds of BPDS are also not prevalent, e.g. with β-alanine (Liao et al., 2001), but the bis(guanidinium) salt is notable as a co-host structure for cooperative guest recognition in clathrate formation with numerous aromatic monocyclic and polycyclic hydrocarbons (Swift & Ward, 1998; Swift et al., 1998; Holman & Ward, 2000).

With compound (I) (Fig. 1), the BPDS dianions lie across crystallographic inversion centres with the sulfonate groups interacting head-to-head through centrosymmetric cyclic bis(water)-bridged hydrogen-bonding associations [graph set R_4_^4^(11) (Etter et al., 1990)], forming one-dimensional chain structures (Fig 2). The cations are linked to these chains through pyridinium N^+^–H···O_water_ hydrogen bonds (Table 1). The second water molecule (O2W) which has only 50% occupancy, forms a O_sulfonate_···H–O–H···O_o__-nitro_ hydrogen bond, bridging the chains down the b axial direction, giving a two-dimensional network structure. There are also weak cation–anion π–π aromatic ring interactions present [minimum ring centroid separation 3.8441 (13) Å]. The hydrogen-bond-constrained o-nitro group in the DNBPY cation in the structure obviates any possible photochromic effects in this compound.

Also present in the BPDS dianions are short intramolecular H2A···H6A^iii^/H6A···H2A^iii^ contacts (2.01 Å) [symmetry code (iii) -x + 2, -y + 1, -z +1] resulting from the BPDS species being planar. There is also a short intramolecular H···H contact involving an aromatic ring H and one of the water H atoms [H6···H22W^i^, 2.06 Å]. With the DNBP cation the associated o-nitro group is rotated out of the plane of the benzene ring while the unassociated p-nitro group is essentially coplanar [torsion angles C11–C21–N21–O22, 149.17 (19)° and C31–C41–N41–O42, 178.02 (9)°].

### Experimental

The title compound was synthesized by heating together under reflux for 10 minutes, 1 mmol quantities of 2-(2,4-dinitrobenzyl)pyridine with biphenyl-4,4'-disulfonic acid in 50 ml of 50% ethanol–water. After concentration to *ca.* 30 ml, partial room temperature evaporation of the hot-filtered solution gave colourless blade-shaped flat prisms (m.p. 413 K) from which a block section was cleaved for the X-ray analysis.

### Refinement

Hydrogen atoms involved in hydrogen-bonding interactions were located by difference methods and their positional and isotropic displacement parameters were refined. Other H atoms were included in the refinement at calculated positions [C–H = 0.93 Å (aromatic) and 0.97 Å (aliphatic) and with *U*_iso_(H) = 1.2*U*_eq_(C)], and treated as riding. One of the water molecules was found to have partial occupancy which was refined to 0.50 (1) and subsequently set invariant.

### Crystal data

**Table:**

2C_12_H_10_N_3_O_4_^+^·C_12_H_8_O_6_S_2_^2^^−^·3H_2_O	*Z* = 1
*M**_r_* = 886.83	*F*(000) = 460
Triclinic, *P*1	*D*_x_ = 1.557 Mg m^−^^3^
Hall symbol: -P 1	Melting point: 413 K
*a* = 8.3897 (3) Å	Mo *K*α radiation, λ = 0.71073 Å
*b* = 10.6455 (4) Å	Cell parameters from 5908 reflections
*c* = 11.7405 (5) Å	θ = 3.0–32.3°
α = 97.879 (3)°	µ = 0.23 mm^−^^1^
β = 96.926 (3)°	*T* = 173 K
γ = 112.066 (4)°	Prism, colourless
*V* = 945.53 (7) Å^3^	0.30 × 0.25 × 0.15 mm

### Data collection

**Table:**

Oxford Diffraction Gemini-S CCD-detector diffractometer	3844 independent reflections
Radiation source: Enhance (Mo) X-ray source	3441 reflections with *I* > 2σ(*I*)
graphite	*R*_int_ = 0.020
Detector resolution: 16.08 pixels mm^-1^	θ_max_ = 26.5°, θ_min_ = 3.0°
ω scans	*h* = −10→10
Absorption correction: multi-scan (*SADABS*; Sheldrick, 1996)	*k* = −13→13
*T*_min_ = 0.98, *T*_max_ = 0.99	*l* = −14→14
8964 measured reflections	

### Refinement

**Table:**

Refinement on *F*^2^	Primary atom site location: structure-invariant direct methods
Least-squares matrix: full	Secondary atom site location: difference Fourier map
*R*[*F*^2^ > 2σ(*F*^2^)] = 0.042	Hydrogen site location: inferred from neighbouring sites
*wR*(*F*^2^) = 0.104	H atoms treated by a mixture of independent and constrained refinement
*S* = 1.03	*w* = 1/[σ^2^(*F*_o_^2^) + (0.0505*P*)^2^ + 0.4454*P*] where *P* = (*F*_o_^2^ + 2*F*_c_^2^)/3
3844 reflections	(Δ/σ)_max_ = 0.001
296 parameters	Δρ_max_ = 0.35 e Å^−^^3^
0 restraints	Δρ_min_ = −0.30 e Å^−^^3^

### Special details

**Table:**

Geometry. Bond distances, angles etc. have been calculated using the rounded fractional coordinates. All su's are estimated from the variances of the (full) variance-covariance matrix. The cell esds are taken into account in the estimation of distances, angles and torsion angles
Refinement. Refinement of *F*^2^ against ALL reflections. The weighted *R*-factor wR and goodness of fit *S* are based on *F*^2^, conventional *R*-factors *R* are based on *F*, with *F* set to zero for negative *F*^2^. The threshold expression of *F*^2^ > σ(*F*^2^) is used only for calculating *R*-factors(gt) etc. and is not relevant to the choice of reflections for refinement. *R*-factors based on *F*^2^ are statistically about twice as large as those based on *F*, and *R*- factors based on ALL data will be even larger.

### Fractional atomic coordinates and isotropic or equivalent isotropic displacement parameters (Å^2^)

**Table:**

	*x*	*y*	*z*	*U*_iso_*/*U*_eq_	Occ. (<1)
O21	0.5436 (2)	0.07535 (18)	0.71892 (15)	0.0487 (6)	
O22	0.51554 (19)	0.13704 (18)	0.55354 (14)	0.0460 (5)	
O41	0.9552 (2)	0.10842 (19)	0.32892 (14)	0.0548 (6)	
O42	1.2220 (2)	0.1865 (2)	0.41565 (15)	0.0627 (7)	
N1	0.7507 (2)	0.15288 (18)	1.02586 (14)	0.0291 (5)	
N21	0.6039 (2)	0.12314 (17)	0.63793 (15)	0.0319 (5)	
N41	1.0680 (2)	0.15615 (18)	0.41645 (15)	0.0350 (5)	
C2	0.8078 (2)	0.1261 (2)	0.92656 (16)	0.0276 (5)	
C3	0.8089 (3)	−0.0026 (2)	0.89375 (18)	0.0348 (6)	
C4	0.7505 (3)	−0.1008 (2)	0.9630 (2)	0.0403 (7)	
C5	0.6935 (3)	−0.0690 (2)	1.06403 (19)	0.0400 (7)	
C6	0.6954 (3)	0.0601 (2)	1.09439 (18)	0.0364 (7)	
C11	0.9122 (2)	0.21211 (18)	0.74513 (16)	0.0256 (5)	
C21	0.7921 (2)	0.16202 (18)	0.63956 (16)	0.0260 (5)	
C31	0.8394 (2)	0.14506 (19)	0.53166 (17)	0.0278 (5)	
C41	1.0145 (2)	0.17560 (19)	0.53089 (16)	0.0275 (5)	
C51	1.1393 (2)	0.2235 (2)	0.63196 (17)	0.0303 (6)	
C61	1.0871 (2)	0.2431 (2)	0.73779 (17)	0.0291 (6)	
C71	0.8663 (3)	0.2446 (2)	0.86313 (17)	0.0318 (6)	
S4A	0.60027 (6)	0.49714 (5)	0.81725 (4)	0.0279 (2)	
O41A	0.71609 (19)	0.57569 (16)	0.92704 (12)	0.0391 (5)	
O42A	0.51335 (19)	0.35151 (15)	0.81872 (14)	0.0412 (5)	
O43A	0.4786 (2)	0.55635 (18)	0.77623 (13)	0.0436 (5)	
C1A	0.9443 (2)	0.50258 (19)	0.54441 (16)	0.0272 (6)	
C2A	0.7662 (3)	0.4645 (3)	0.51220 (19)	0.0576 (9)	
C3A	0.6617 (3)	0.4658 (3)	0.59494 (19)	0.0531 (9)	
C4A	0.7357 (2)	0.50677 (19)	0.71157 (16)	0.0263 (6)	
C5A	0.9122 (3)	0.5469 (2)	0.74546 (18)	0.0399 (6)	
C6A	1.0155 (3)	0.5444 (2)	0.66214 (19)	0.0409 (7)	
O1W	0.7300 (2)	0.39624 (19)	1.07848 (14)	0.0422 (6)	
O2W	0.4510 (4)	0.7847 (3)	0.7040 (3)	0.0475 (11)	0.500
H1	0.746 (4)	0.240 (3)	1.049 (2)	0.059 (8)*	
H3	0.84830	−0.02380	0.82590	0.0420*	
H4	0.75000	−0.18830	0.94090	0.0480*	
H5	0.65440	−0.13420	1.11060	0.0480*	
H6	0.65840	0.08380	1.16260	0.0440*	
H31	0.75700	0.11440	0.46260	0.0330*	
H51	1.25620	0.24210	0.62900	0.0360*	
H61	1.17110	0.27820	0.80620	0.0350*	
H71	0.96790	0.31920	0.91260	0.0380*	
H72	0.77380	0.27800	0.85240	0.0380*	
H2A	0.71510	0.43730	0.43320	0.0690*	
H3A	0.54180	0.43900	0.57140	0.0640*	
H5A	0.96300	0.57590	0.82450	0.0480*	
H6A	1.13540	0.57160	0.68620	0.0490*	
H11W	0.662 (4)	0.407 (3)	1.127 (3)	0.069 (9)*	
H12W	0.719 (4)	0.441 (3)	1.031 (3)	0.062 (9)*	
H21W	0.460 (5)	0.730 (4)	0.745 (4)	0.060 (10)*	0.500
H22W	0.515 (5)	0.860 (3)	0.755 (3)	0.065 (10)*	0.500

### Atomic displacement parameters (Å^2^)

**Table:**

	*U*^11^	*U*^22^	*U*^33^	*U*^12^	*U*^13^	*U*^23^
O21	0.0343 (8)	0.0630 (11)	0.0535 (10)	0.0155 (8)	0.0225 (7)	0.0261 (8)
O22	0.0318 (8)	0.0596 (10)	0.0475 (9)	0.0198 (8)	0.0040 (7)	0.0119 (8)
O41	0.0577 (11)	0.0678 (12)	0.0295 (8)	0.0162 (9)	0.0131 (8)	0.0011 (8)
O42	0.0491 (10)	0.1108 (16)	0.0475 (10)	0.0453 (11)	0.0280 (8)	0.0225 (10)
N1	0.0249 (8)	0.0370 (10)	0.0239 (8)	0.0107 (7)	0.0051 (6)	0.0052 (7)
N21	0.0282 (9)	0.0288 (9)	0.0387 (9)	0.0105 (7)	0.0107 (7)	0.0053 (7)
N41	0.0446 (10)	0.0357 (9)	0.0341 (9)	0.0210 (8)	0.0187 (8)	0.0131 (8)
C2	0.0229 (9)	0.0318 (10)	0.0246 (9)	0.0074 (8)	0.0048 (7)	0.0042 (8)
C3	0.0401 (11)	0.0317 (11)	0.0320 (10)	0.0133 (9)	0.0092 (9)	0.0057 (8)
C4	0.0397 (12)	0.0326 (11)	0.0442 (12)	0.0116 (10)	−0.0008 (9)	0.0097 (9)
C5	0.0294 (11)	0.0508 (13)	0.0386 (12)	0.0104 (10)	0.0044 (9)	0.0236 (10)
C6	0.0273 (10)	0.0550 (14)	0.0270 (10)	0.0136 (10)	0.0067 (8)	0.0152 (9)
C11	0.0304 (10)	0.0207 (9)	0.0275 (9)	0.0096 (8)	0.0115 (7)	0.0068 (7)
C21	0.0253 (9)	0.0213 (9)	0.0334 (10)	0.0094 (7)	0.0102 (7)	0.0075 (7)
C31	0.0301 (10)	0.0254 (9)	0.0275 (9)	0.0106 (8)	0.0058 (7)	0.0054 (7)
C41	0.0331 (10)	0.0259 (9)	0.0287 (9)	0.0140 (8)	0.0134 (8)	0.0090 (8)
C51	0.0265 (10)	0.0333 (10)	0.0366 (11)	0.0141 (8)	0.0132 (8)	0.0116 (8)
C61	0.0286 (10)	0.0302 (10)	0.0286 (10)	0.0115 (8)	0.0051 (8)	0.0072 (8)
C71	0.0381 (11)	0.0269 (10)	0.0294 (10)	0.0106 (9)	0.0131 (8)	0.0034 (8)
S4A	0.0314 (3)	0.0369 (3)	0.0266 (2)	0.0204 (2)	0.0159 (2)	0.0132 (2)
O41A	0.0427 (8)	0.0521 (9)	0.0272 (7)	0.0214 (7)	0.0150 (6)	0.0081 (6)
O42A	0.0408 (8)	0.0418 (9)	0.0492 (9)	0.0168 (7)	0.0242 (7)	0.0205 (7)
O43A	0.0532 (9)	0.0702 (11)	0.0371 (8)	0.0471 (9)	0.0254 (7)	0.0254 (8)
C1A	0.0332 (10)	0.0285 (10)	0.0298 (10)	0.0170 (8)	0.0174 (8)	0.0136 (8)
C2A	0.0474 (14)	0.127 (2)	0.0247 (11)	0.0570 (16)	0.0166 (10)	0.0264 (13)
C3A	0.0385 (12)	0.113 (2)	0.0303 (11)	0.0483 (14)	0.0158 (10)	0.0256 (13)
C4A	0.0325 (10)	0.0283 (10)	0.0295 (9)	0.0187 (8)	0.0174 (8)	0.0129 (8)
C5A	0.0303 (10)	0.0487 (13)	0.0278 (10)	0.0045 (9)	0.0116 (8)	−0.0068 (9)
C6A	0.0241 (10)	0.0476 (13)	0.0370 (11)	0.0014 (9)	0.0144 (8)	−0.0061 (10)
O1W	0.0555 (10)	0.0677 (11)	0.0272 (8)	0.0456 (9)	0.0177 (7)	0.0154 (8)
O2W	0.0471 (19)	0.0426 (18)	0.0560 (15)	0.0206 (15)	0.0161 (15)	0.0066 (15)

### Geometric parameters (Å, °)

**Table:**

S4A—O42A	1.4479 (16)	C21—C31	1.381 (3)
S4A—O43A	1.4558 (19)	C31—C41	1.382 (3)
S4A—C4A	1.7687 (19)	C41—C51	1.378 (3)
S4A—O41A	1.4481 (15)	C51—C61	1.381 (3)
O21—N21	1.214 (2)	C3—H3	0.9300
O22—N21	1.224 (2)	C4—H4	0.9300
O41—N41	1.213 (2)	C5—H5	0.9300
O42—N41	1.210 (3)	C6—H6	0.9300
O1W—H11W	0.88 (4)	C31—H31	0.9300
O1W—H12W	0.80 (3)	C51—H51	0.9300
O2W—H21W	0.82 (4)	C61—H61	0.9300
O2W—H22W	0.87 (3)	C71—H71	0.9700
N1—C6	1.342 (3)	C71—H72	0.9700
N1—C2	1.348 (2)	C1A—C2A	1.381 (3)
N21—C21	1.471 (3)	C1A—C6A	1.378 (3)
N41—C41	1.479 (3)	C1A—C1A^i^	1.491 (3)
N1—H1	0.95 (3)	C2A—C3A	1.387 (4)
C2—C3	1.375 (3)	C3A—C4A	1.371 (3)
C2—C71	1.506 (3)	C4A—C5A	1.367 (3)
C3—C4	1.392 (3)	C5A—C6A	1.387 (3)
C4—C5	1.378 (3)	C2A—H2A	0.9300
C5—C6	1.365 (3)	C3A—H3A	0.9300
C11—C61	1.394 (3)	C5A—H5A	0.9300
C11—C71	1.512 (3)	C6A—H6A	0.9300
C11—C21	1.396 (3)		
			
O43A—S4A—C4A	105.78 (9)	C2—C3—H3	120.00
O41A—S4A—C4A	106.12 (9)	C4—C3—H3	120.00
O41A—S4A—O42A	112.56 (9)	C5—C4—H4	120.00
O41A—S4A—O43A	113.37 (10)	C3—C4—H4	120.00
O42A—S4A—O43A	112.51 (10)	C6—C5—H5	121.00
O42A—S4A—C4A	105.74 (9)	C4—C5—H5	121.00
H11W—O1W—H12W	104 (3)	N1—C6—H6	120.00
H21W—O2W—H22W	97 (4)	C5—C6—H6	120.00
C2—N1—C6	123.13 (19)	C41—C31—H31	121.00
O21—N21—O22	123.47 (19)	C21—C31—H31	121.00
O22—N21—C21	118.33 (17)	C61—C51—H51	121.00
O21—N21—C21	118.17 (17)	C41—C51—H51	121.00
O42—N41—C41	118.02 (17)	C11—C61—H61	119.00
O41—N41—O42	123.59 (19)	C51—C61—H61	119.00
O41—N41—C41	118.38 (18)	H71—C71—H72	107.00
C6—N1—H1	117.1 (16)	C2—C71—H72	108.00
C2—N1—H1	119.8 (16)	C11—C71—H71	108.00
N1—C2—C3	118.39 (18)	C11—C71—H72	108.00
N1—C2—C71	114.88 (18)	C2—C71—H71	108.00
C3—C2—C71	126.73 (18)	C1A^i^—C1A—C2A	121.47 (17)
C2—C3—C4	119.4 (2)	C1A^i^—C1A—C6A	121.02 (18)
C3—C4—C5	120.3 (2)	C2A—C1A—C6A	117.51 (19)
C4—C5—C6	118.71 (19)	C1A—C2A—C3A	121.5 (2)
N1—C6—C5	120.0 (2)	C2A—C3A—C4A	119.8 (2)
C61—C11—C71	118.98 (18)	S4A—C4A—C5A	120.50 (15)
C21—C11—C71	124.29 (18)	C3A—C4A—C5A	119.8 (2)
C21—C11—C61	116.55 (17)	S4A—C4A—C3A	119.64 (17)
C11—C21—C31	123.34 (17)	C4A—C5A—C6A	120.1 (2)
N21—C21—C11	120.82 (16)	C1A—C6A—C5A	121.4 (2)
N21—C21—C31	115.84 (16)	C1A—C2A—H2A	119.00
C21—C31—C41	117.06 (17)	C3A—C2A—H2A	119.00
N41—C41—C51	119.43 (17)	C4A—C3A—H3A	120.00
C31—C41—C51	122.50 (17)	C2A—C3A—H3A	120.00
N41—C41—C31	118.07 (17)	C4A—C5A—H5A	120.00
C41—C51—C61	118.54 (17)	C6A—C5A—H5A	120.00
C11—C61—C51	121.97 (18)	C1A—C6A—H6A	119.00
C2—C71—C11	115.69 (17)	C5A—C6A—H6A	119.00
			
O43A—S4A—C4A—C5A	137.52 (17)	C61—C11—C21—C31	−1.2 (3)
O42A—S4A—C4A—C3A	73.2 (2)	C71—C11—C21—N21	−6.5 (3)
O41A—S4A—C4A—C3A	−167.09 (19)	C71—C11—C21—C31	173.82 (18)
O41A—S4A—C4A—C5A	16.81 (19)	C21—C11—C61—C51	−1.0 (3)
O42A—S4A—C4A—C5A	−102.95 (18)	C71—C11—C61—C51	−176.22 (18)
O43A—S4A—C4A—C3A	−46.4 (2)	N21—C21—C31—C41	−177.51 (17)
C6—N1—C2—C71	−179.2 (2)	C11—C21—C31—C41	2.2 (3)
C6—N1—C2—C3	0.2 (3)	C21—C31—C41—N41	179.06 (17)
C2—N1—C6—C5	−0.7 (3)	C21—C31—C41—C51	−1.2 (3)
O21—N21—C21—C11	−32.8 (3)	C31—C41—C51—C61	−0.8 (3)
O21—N21—C21—C31	146.90 (19)	N41—C41—C51—C61	178.96 (18)
O22—N21—C21—C31	−31.1 (3)	C41—C51—C61—C11	1.9 (3)
O22—N21—C21—C11	149.17 (19)	C6A—C1A—C2A—C3A	−1.1 (4)
O42—N41—C41—C31	178.02 (19)	C1A^i^—C1A—C2A—C3A	178.3 (2)
O42—N41—C41—C51	−1.7 (3)	C2A—C1A—C6A—C5A	0.7 (3)
O41—N41—C41—C51	176.96 (19)	C1A^i^—C1A—C6A—C5A	−178.72 (19)
O41—N41—C41—C31	−3.3 (3)	C2A—C1A—C1A^i^—C2A^i^	−180.0 (2)
N1—C2—C71—C11	−173.63 (18)	C2A—C1A—C1A^i^—C6A^i^	0.7 (3)
N1—C2—C3—C4	0.4 (3)	C6A—C1A—C1A^i^—C2A^i^	−0.7 (3)
C71—C2—C3—C4	179.8 (2)	C6A—C1A—C1A^i^—C6A^i^	180.0 (2)
C3—C2—C71—C11	7.0 (3)	C1A—C2A—C3A—C4A	0.6 (4)
C2—C3—C4—C5	−0.6 (4)	C2A—C3A—C4A—S4A	−175.7 (2)
C3—C4—C5—C6	0.1 (4)	C2A—C3A—C4A—C5A	0.4 (4)
C4—C5—C6—N1	0.5 (4)	S4A—C4A—C5A—C6A	175.26 (16)
C21—C11—C71—C2	88.6 (2)	C3A—C4A—C5A—C6A	−0.8 (3)
C61—C11—C71—C2	−96.6 (2)	C4A—C5A—C6A—C1A	0.3 (3)
C61—C11—C21—N21	178.55 (17)		

Symmetry codes: (i) −*x*+2, −*y*+1, −*z*+1.

### Hydrogen-bond geometry (Å, °)

**Table:**

*D*—H···*A*	*D*—H	H···*A*	*D*···*A*	*D*—H···*A*
N1—H1···O1W	0.95 (3)	1.71 (3)	2.655 (3)	175 (3)
O1W—H11W···O43A^ii^	0.88 (4)	1.84 (4)	2.716 (2)	175 (3)
O1W—H12W···O41A	0.80 (3)	2.01 (3)	2.806 (2)	172 (3)
O2W—H21W···O43A	0.82 (4)	1.99 (4)	2.761 (4)	155 (4)
O2W—H22W···O21^iii^	0.87 (3)	2.32 (3)	2.867 (2)	124 (3)
C2A—H2A···O2W^iv^	0.93	2.46	3.195 (4)	136
C4—H4···O41A^v^	0.93	2.40	3.309 (3)	165
C5—H5···O42A^vi^	0.93	2.53	3.427 (3)	163
C5A—H5A···O41A	0.93	2.52	2.897 (3)	105
C5A—H5A···O1W^vii^	0.93	2.58	3.232 (3)	128
C6—H6···O2W^ii^	0.93	2.44	3.316 (4)	156
C6—H6···O21^vi^	0.93	2.60	3.265 (3)	129
C71—H72···O21	0.97	2.46	2.799 (3)	100
C71—H72···O42A	0.97	2.59	3.558 (3)	176

Symmetry codes: (ii) −*x*+1, −*y*+1, −*z*+2; (iii) *x*, *y*+1, *z*; (iv) −*x*+1, −*y*+1, −*z*+1; (v) *x*, *y*−1, *z*; (vi) −*x*+1, −*y*, −*z*+2; (vii) −*x*+2, −*y*+1, −*z*+2.
